# Supplementary material for: Dopamine-derived nitrogen-doped carboxyl multiwalled carbon nanotube-modified graphite felt with improved electrochemical activity for vanadium redox flow batteries
Source: R Soc Open Sci. 2020 Jul 1;7(7):200402. doi: 10.1098/rsos.200402 (PMC7428217; doi:10.1098/rsos.200402)
Supplement: Electronic supplementary material for preparation of MWCNT@PDA [file rsos200402supp1.docx]

**Dopamine-derived nitrogen doped carboxyl MWCNT modified graphite felt with improved electrochemical activity for vanadium redox flow batteries**

Qiang Li,^1^ Anyu Bai,^1^ Tianyu Zhang,^1^ Song Li ^1^ and Hong Sun ^*1^

^1^School of Mechanical Engineering, Shenyang Jianzhu University, Shenyang, China, 110168. Tel: 024-24692171,

^*^**Author for correspondence:** Hong Sun, Email: [sunhongwxh@sina.com](mailto:sunhongwxh@sina.com)

**2.1 Materials and methods**

The procedure for the preparation of MWCNT@PDA was based on the reference [34] and the detail process was shown as follows. Firstly, 0.4 g carboxyl MWCNT were added into 600 mL deionized water. Subsequently, 0.9 g dopamine hydrochloride were uniformly dispersed in the suspension containing carboxyl MWCNT under ultrasonic condition for 1h at room temperature. Then, Tris-HCl buffer solution was slowly dipped into the mixed solution until the pH reaches to 8.5 and the self-polymerization of dopamine process was occurred. The carboxyl MWCNT@PDA were obtained after the self-polymerization of dopamine for 24 h at room temperature. Finally, the as-prepared products were vacuum filtered, washed with deionized water for several times, and dried for further use.

34. Yu L, Lin F, Xiao W, Luo D, Xi J. 2018 CNT@polydopamine embedded mixed matrix membranes for high-rate and long-life vanadium flow batteries. *J. Membrane Sci.* **549**, 411-419. (10.1016/j.memsci.2017.12.043)
